# Identification of a novel splice-site mutation in the *Lebercilin (LCA5)* gene causing Leber congenital amaurosis

**Published:** 2008-03-10

**Authors:** Vedam Lakshmi Ramprasad, Nagasamy Soumittra, Derek Nancarrow, Parveen Sen, Martin McKibbin, Grange A Williams, Tharigopala Arokiasamy, Praveena Lakshmipathy, Chris F Inglehearn, Govindasamy Kumaramanickavel

**Affiliations:** 1 Department of Genetics and Molecular Biology, Vision Research Foundation, Sankara Nethralaya Chennai, India; 2 Oncogenomics, Queensland Institute of Medical Research Foundation, Herston, Queensland, Australia; 3 Department of Medical Retina, Medical Research Foundation, Sankara Nethralaya, Chennai, India; 4 Section of Ophthalmology and Neuroscience, Leeds Institute of Molecular Medicine, St. James’s University Hospital, Leeds, United Kingdom

## Abstract

**Purpose:**

Leber congenital amaurosis (LCA) is one of the most common causes of hereditary blindness in infants. To date, mutations in 13 known genes and at two other loci have been implicated in LCA causation. An examination of the known genes highlights several processes which, when defective, cause LCA, including photoreceptor development and maintenance, phototransduction, vitamin A metabolism, and protein trafficking. In addition, it has been known for some time that defects in sensory cilia can cause syndromes involving hereditary blindness. More recently evidence has come to light that non-syndromic LCA can also be a “ciliopathy.”

**Methods:**

Here we present a homozygosity mapping analysis in a consanguineous sibship that led to the identification of a mutation in the recently discovered *LCA5* gene. Homozygosity mapping was done using Affymetrix 10K Xba I Gene Chip and a 24.5cM region on chromosome 6 (6q12- q16.3) was identified to be significantly homozygous. The *LCA5* gene on this region was sequenced and cDNA sequencing also done to characterize the mutation.

**Results:**

A c.955G>A missense mutation in the last base of exon 6 causing disruption of the splice donor site was identified in both the affected sibs. Since there is a second consensus splice donor sequence 5 bp into the adjacent intron, this mutation results in a transcript with a 5 bp insertion of intronic sequence, leading to a frameshift and premature truncation.

**Conclusions:**

We report a missense mutation functionally altering the splice donor site and leading to a truncated protein. This is the second report of *LCA5* mutations causing LCA. It may also be significant that one affected child died at eleven months of age due to asphyxia during sleep. To date the only phenotype unambiguously associated with mutations in this gene is LCA. However the *LCA5* gene is known to be expressed in nasopharynx, trachea and lungs and was originally identified in the proteome of bronchial epithelium ciliary axonemes. The cause of death in this child may therefore imply that *LCA5* mutations can in fact cause a wider spectrum of phenotypes including respiratory disease.

## Introduction

Retinal dystrophies are a clinically and genetically heterogeneous group of inherited diseases that cause severe visual impairment. Estimated worldwide incidence is 1 in 3000 to 5000, and all three modes of Mendelian inheritance are observed [1]. Leber congenital amaurosis (LCA) is an autosomal recessive condition that appears at birth or in the first few months of life. LCA is typically characterized by nystagmus, sluggish or no pupillary response, and severe vision loss [1]. It is one of the most common genetic causes of congenital visual impairment in infants and children. Genes implicated in LCA causation include retinal guanylate cyclase (*GUCY2D*) on chromosome 17p13.1 (LCA1), *RPE65* on chromosome 1p31 (LCA2), *RDH12* on chromosome 14q23.3 (which may account for the LCA3 locus, though this remains to be clarified), *AIPL1* on chromosome 17p13.1 (LCA4), *RPGRIP1* on chromosome 14q11 (LCA6), *CRX* on chromosome 19q13.3 (LCA7), *CRB1* on chromosome 1q31.3 (LCA8), *CEP290* on chromosome 12q21.3 (LCA10), and *IMPDH1* on chromosome 7q32.1 (LCA11) [2]. Mutations in *LRAT* (4q32.1) and *TULP1* (6p21.31) genes have also been identified in recessive LCA. LCA9 has been mapped to 1p36, but a causative gene has not been identified [2]. The location of LCA5 was mapped to 6q11-q16 [3] and was later confirmed in an independent pedigree [4]. Recent work has demonstrated that the LCA5 causative gene is *lebercilin*, previously known as C6orf152 [5].

In this study we describe a consanguineous nuclear pedigree in two sisters who have LCA. In this family we performed a genome wide screen for homozygosity. A homozygous region was identified at the *LCA5* locus, and a novel mutation was identified in the *LCA5* gene. This is the second report of a mutation in the *LCA5* gene and the fifth mutation reported.

## Methods

### Clinical examination

The research protocol was approved by the ethics review board of the Vision Research Foundation, Sankara Nethralaya. The study procedures were performed in accordance with institutional guidelines and the Declaration of Helsinki, and informed consent was obtained from each participant. The two affected sisters, their unaffected parents aged 30 (mother) and 36 years (father) and 50 ethnically matched controls, which included 28 males and 22 females with an average age of 62.32 (ranging between 38 and 79 years), underwent a detailed ophthalmic examination including recording of best corrected visual acuity, refraction, slit lamp examination, and post dilatation binocular indirect ophthalmoscopy. Additionally electroretinography was done on patients.

### DNA extraction

We drew 10 ml of heparinized blood from both affected patients and their parents after obtaining informed consent from both parents. 10 ml of blood was also drawn from 50 ethnically matched controls after obtaining informed consent. Genomic DNA was extracted immediately using QIAamp Blood DNA maxi kit (Qiagen, GmbH, Germany) according to the manufacturer’s instructions. Also consent was obtained from the parents on behalf of the patients because of their underage.

### Genotyping

Single nucleotide polymorphism (SNP) genotyping was performed with the GeneChip Mapping 10K Xba I Array and Assay Kit (Affymetrix, Santa Clara, CA). All incubations were done using a GeneAmp PCR system 9700 (Applied Biosystems, Foster City, CA). Internal positive and negative GeneChip controls were performed in parallel using the supplied genomic DNA. Xba I (New England Biolabs, Ipswich, MA) was used to digest 250 ng genomic DNA for 2 h at 37 °C followed by heat inactivation for 20 min at 70 °C. Digested DNA was then incubated with a 0.25 M Xba I adaptor (Affymetrix) and DNA ligase (New England Biolabs) in standard ligation buffer for 2 h at 16 °C followed by heat inactivation for 20 min at 70 °C. Ligated products were amplified in quadruplicate using 10 μM generic primer in PCR buffer II (Applied Biosystems) with 2.5 mM MgCl_2_/2.5 mM deoxyribose nucleotide triphosphates (dNTPs)/10 units of AmpliTaq Gold polymerase (Applied Biosystems) under the following PCR conditions: 95 °C for 5 min, followed by 35 cycles (95 °C for 20 s, 59 °C for 15 s, and 72 °C for 15 s) and a final extension at 72 °C for 7 min. Fragments ranging in size from 250 to 1,000 bp were preferentially amplified under the conditions [6]. PCR products were purified with Qiagen MinElute 96 UF PCR Purification Kit and concentrated with a Qiagen PCR purification column (Qiagen,) according to the manufacturer's recommendations. A 10K genotyping assay kit fragmentation reagent (Affymetrix) was used to digest 20 μg of DNA, which was then labeled with 30 U/μL terminal deoxynucleotidyl transferase and 5 mM DNA labeling reagent (Affymetrix 10K genotyping assay kit). After undergoing heat inactivation at 95 °C for 10 min, samples were injected into microarray cartridges and hybridized overnight. Microarrays were washed in a fluidics station 450 (Affymetrix), followed by staining with streptavidin Avidin Phycoerythrin (Molecular Probes, Eugene, OR), and biotinylated antistreptavidin (Vector Lab, Burlingame, CA), followed by a final wash with SSPE buffer. Microarrays were scanned according to manufacturer’s directions (Affymetrix). The data was analysed using Exclude AR program (ExcludeAR sheet; Excel, Microsoft, Redmond, WA) [7].

### Sequencing of the *LCA5* gene

The seven coding exons of the *LCA5* gene were amplified using 11 sets of primers with exon 7 amplified using five sets of overlapping primers (same primers used by den Hollander et al. [5]). A 20 ml reaction was set up containing 10 mM Tris (pH 9.0), 50 mM KCl, 1.5mM MgCl_2_ and 0.01% gelatin, 1 mM dNTP each (GeNei, Bangalore, India), 10 mM of each forward and reverse primer, 1U of Taq DNA polymerase (GeNei, Bangalore, India) and 5 mM betaine (Sigma Aldrich, St. Louis, MO). 100 ng of genomic DNA was amplified with initial denaturation at 94°C for 5 min followed by 35 cycles of denaturation at 94 °C for 20 s, annealing at (56 °C for exons 1, 2a, 2b, 2c, 5, 6, 7e, 57 °C for exon 7a, 7b, 7c, 60 °C for 7d, and at 65-58/58 touchdown for exon 3 and 4) for 20 s and extension at 72 °C for 45 s and final extension at 72 °C for 7 min. PCR products were digested with exonuclease I, *E. coli*, and shrimp alkaline phosphatase (Fermentas Life Sciences, Glen Burnie, MD) sequenced unidirectionally using BigDye Terminator v.3.1 kit (Applied Biosystems) with specific primers in ABI3100 Avant, (Applied Biosystems). The sequences were analyzed in Sequence Analysis software v 3.1.1. (Applied Biosystems, Foster City, CA). Any DNA sequence variations were confirmed in the reverse direction. Fifty ethnically matched normal controls were also amplified and sequenced to confirm the mutation.

### In silico splice site prediction

The effect of the single base substitution identified in the last base position of exon 6 was evaluated using a splice site prediction algorithm (http://violin.genet.sickkids.on.ca/~ali/splicesitefinder.html) [8,9].

### RNA isolation and cDNA sequencing

RNA was isolated from the lymphocytes separated from 10-ml heparinized blood samples of the affected (proband), unaffected parents, and one unrelated normal control by using Trizol reagent (Sigma-Aldrich, St. Louis, MO), according to the manufacturer’s instructions, and dissolved in diethyl pyrocarbonate (DEPC)–treated water. Total RNA was used to generate a cDNA pool by RT–PCR using a Qiagen Sensiscript reverse transcriptase kit (Qiagen, GmbH, Germany) according to the manufacturer’s instructions (Qiagen). PCR primers [10] for the *GAPDH* housekeeping gene were used as the internal control. For the amplification of the *LCA5* gene, exonic primers were used spanning exons 5, 6, and 7 (forward GCTGAAAGGAAAAGGGCATA and reverse GGCTTGAAGTCTTCCATGGTT). PCR amplification was performed using 50 ng of cDNA, 10 mM Tris (pH 9.0), 50 mM KCl, 1.5 mM MgCl_2_ and 0.01% gelatin, 1 mM dNTP each (GeNei, Bangalore, India), 10 μM each of forward and reverse primer, 1U of Taq DNA polymerase (GeNei) and 5 mM betaine (Sigma Aldrich) at 94 °C for 5 min followed by 35 cycles of 94 °C for 30 s, 57 °C for 45 s, 72 °C for 1 min, and a final extension of 72 °C for 7 min.

## Results

**Figure 1:**
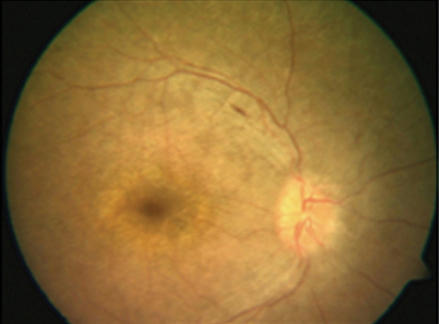
Color fundus photograph of the right eye. Fundus photograph of the right eye of the proband showing midperipheral white dots at the level of the retinal pigment epithelium, arteriolar attenuation and an abnormal sheen in the macula.

**Figure 2:**
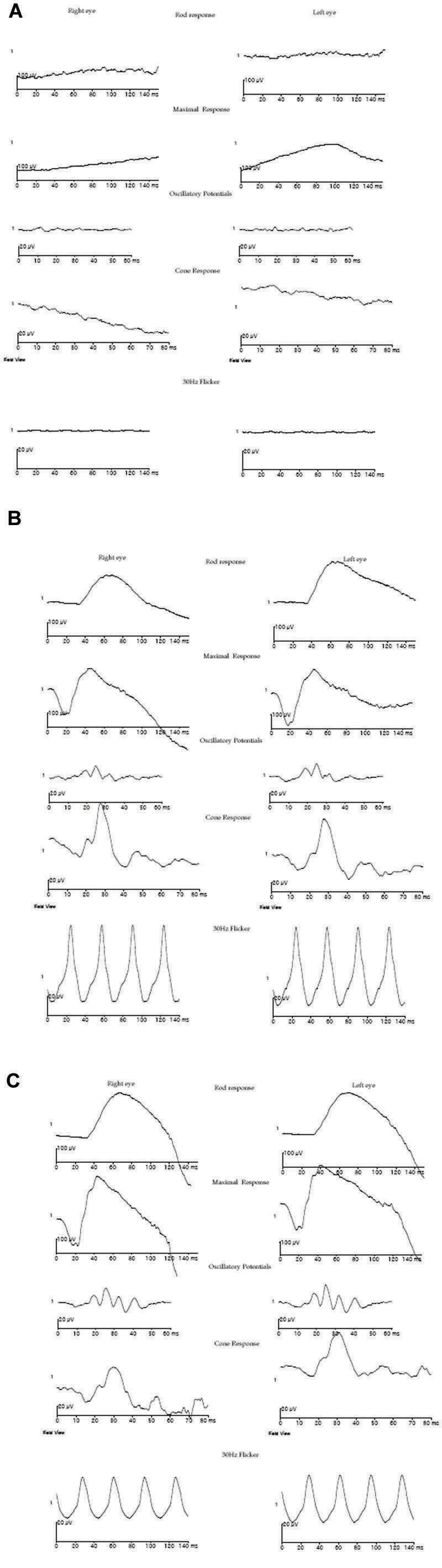
Full field electroretinogram in patient and parents. Full field electroretinogram (ERG) of the proband (**A**), unaffected father (**B**), and unaffected mother (**C**). The ERG is normal for the parents but severely attenuated for the proband.

A 7-month-old female of Indian ancestry presented with a history of not following or fixating on a light source. Searching nystagmus and oculodigital reflex were present. Bilateral high hyperopia was observed, with cycloplegic refraction of +9.50 diopter sphere (DS)-5.00 diopter cylinder (DC)×180° in the right eye and +7.00DS-3.00DC×180° in the left eye. While anterior segment evaluation was normal, fundus examination revealed diffuse, bilateral retinal pigmentary abnormalities of pepper and salt type, arteriolar attenuation, and a metallic sheen. Optic discs appeared normal. A diagnosis of LCA was confirmed when the photopic and scotopic electroretinogram (ERG) responses were found to be nonrecordable, implicating the involvement of both cone and rod photoreceptors. Reexamination of the child when she was 6 years old revealed similar findings of high hyperopia, nystagmus, retinal pigmentary alterations, including white dots in the mid-periphery and an abnormal sheen in the macula (Figure 1), and an essentially nonrecordable ERG (Figure 2A), suggestive of LCA. However, no history of mental retardation, cystic renal disease, skeletal disorders, hydrocephalus or any other systemic associations were noted. No systemic complications were seen on examination.

The proband’s younger sister (younger by 6 years) was examined at the age of 5 months. She presented to the hospital with poor vision and rotatory movements of the eyeball. On examination the child was seen to follow light with poor fixation. Searching nystagmus was present along with bilateral high hyperopia. Cycloplegic refraction was +6.50DS-1.50DC×180° in the right eye and +8.25DS-1.50DC×170° in the left eye. Anterior segment evaluation was normal, but as for the proband (old sister), fundus examination revealed retinal pigmentary alterations of pepper and salt type, mild arteriolar attenuation and a metallic sheen. Six months after the examination the younger sister died due to asphyxia during sleep.

Both parents had a normal visual function and normal retina. Given that the parents were second cousins, and that the two sisters presented with similar symptoms, which were not reported in any other family members, we assigned an autosomal recessive inheritance of LCA.

DNA from the two affected sisters and the unaffected parents was hybridized onto GeneChips that were then scanned. Data analysis using Exclude AR program identified two significant homozygously shared regions between the two affected sisters (data not shown). The first was a 24.5 cM region on chromosome 6 (6q12-q16.3) made up of 139 consecutive SNPs, and the other was a 24.1 cM region on chromosome 7 (7q21.1-q22.3) that was composed of 83 consecutive SNPs. Given the prior linkage to chromosome 6q in LCA5 [3,4] we sequenced the *LCA5* gene [5] in all four pedigree members.

Sequence analysis of the *LCA5* gene at 6q14.1 revealed variations in exons 3 and 6. In exon 3 we noted a T>C polymorphism (c.71T>C, known SNP rs2655655), resulting in a Lys24Ser substitution, with the affected siblings and carrier parents all homozygous for the C allele. Also in exon 3, an A>C (c.77A>C, rs34068461) polymorphism, leading to p.Asp26Ala, was seen in heterozygous form in the father and as a homozygous change in the remaining three family members.

**Figure 3:**
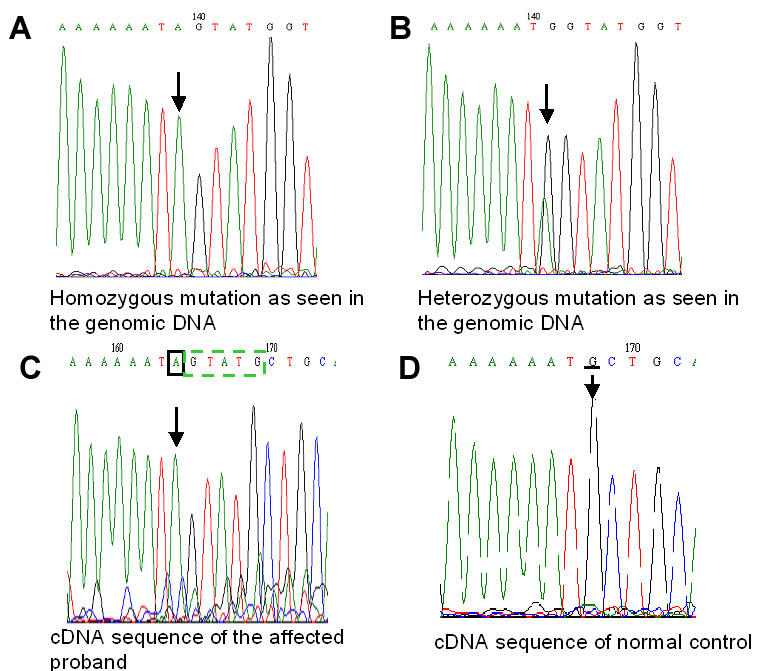
Mutation analysis of *LCA5* gene. **A:** Sequence chromatogram of the *LCA5* gene showing c.955G>A homozygous mutation in the genomic DNA of the affected patient. The homozygous mutation is indicated by the arrow. **B:** Sequence chromatogram of the *LCA5* gene showing c.955G>A heterozygous change in the genomic DNA of the unaffected father. The heterozygous variation is indicated by the arrow. **C:** cDNA sequence of the *LCA5* gene of the affected proband with the mutated splice site. The black square box and the arrow indicate the mutated base. The green dashed box indicates the 5 base insertion of the adjacent intron due to the donor splice site mutation. **D:** cDNA sequence of a normal control showing the wild type base as indicated by the arrow and the underlined sequence annotation.

**Figure 4:**
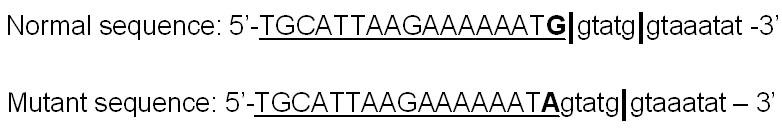
Schematic representation of the 3' end of the exon 6 of the *LCA5* gene. The figure represents 3' portion of the normal and mutant sequence of the exon 6 of the *LCA5* gene. Nucleotides in uppercase represent exonic sequence and that in small lower case represent intronic sequences. The nucleotide in uppercase and in bold represent the last base of the exon and the site of mutation. The first vertical bar in the normal sequence represent the real splice donor site and the second vertical bar represent additional/alternative splice donor site, which is activated in the event of absence of the real splice donor site.

In addition, a G>A variation was identified (c.955G>A) in the last base of exon 6, which would be expected to cause a p.Ala319Thr missense mutation. This mutation was homozygous in the affected siblings (Figure 3A) while both parents were heterozygous carriers (Figure 3B). This change was not seen in 50 normal controls of a similar ethnic background. As it affected the last base of exon 6, we used a splice site prediction algorithm [8,9] to investigate the possibility this mutation might also lead to aberrant mRNA splicing. The algorithm calculates scores for potential donor and acceptor sequences that provide an estimate of the strength of these sequences as sites for initiation of splicing. Our analysis of the normal sequence adjacent to the 3' end of exon 6 predicted two donor sites that turned out to be the wild-type exon 6 donor-site and a second donor-site 5 bp into the downstream intron. The algorithm scored these sequences equally as potential donor sites (76.3 and 77.7% respectively). However, when the mutated sequence was tested, the original donor site was no longer predicted. This analysis suggested that the mutation might cause exon 6 to be spliced at the alternative donor site, producing an mRNA with a 5 bp insertion of the intron, breaking the reading frame and potentially truncating the protein (Figure 4).

To test this hypothesis, we amplified cDNA spanning exons 5, 6, and 7 of the *LCA5* gene from the affected proband and sequenced. The results obtained were as predicted. The proband was found to have a 5 bp insertion of intronic sequence (Figure 3C), causing a frameshift in the mRNA, while the control showed the normal sequence (Figure 3D). The c.955G>A substitution mutation is therefore not a missense mutation but a nonsense mutation, which will lead either to insertion of 29 new amino acids sequence before premature truncation.

## Discussion

Recently den Hollander et al. [5] demonstrated that the defective gene at the *LCA5* locus encodes the ciliary protein lebercilin [3]. They identified one nonsense mutation, two frameshift mutations, and one promoter mutation in consanguineous LCA families [5]. Here we used homozygosity mapping in a consanguineous Indian pedigree to identify a novel *LCA5* mutation, c.955G>A, that disrupts the correct exon 6 splice donor site and leads to splicing at a cryptic donor consensus sequence 5 bp into the adjacent intron. This finding further underlines the importance of homozygosity mapping as a tool for identifying genes and mutations involved in recessively inherited diseases, and of nonsense mutations in the *LCA5* gene as a cause of LCA. The mutation identified is interesting as it serves to further emphasize that defects in splicing, as well as direct alterations of the protein code, can cause human inherited diseases. A similar change in the third base of a codon could easily be overlooked in such analyses since at first glance it is a silent change in terms of its effect on the mRNA code.

The phenotype of the family described herein is consistent with a diagnosis of LCA and is similar to the phenotype described previously in LCA5 patients of the same age [4]. Despite the fact that LCA is a congenital abnormality, the previous report suggested some progression of phenotype with age, with macular staphyloma as a complication of disease in adulthood. The two patients observed in this report were both below ten years of age on examination and had no staphylomatous changes. The relatively consistent LCA5 phenotype, the recessive mode of inheritance, and the growing list of null mutations all point to the LCA5 phenotype being the result of a lack of functional lebercilin protein rather than the presence of a defective protein. It is not yet known whether truncated proteins are produced in patients or whether the mutated mRNAs are degraded by nonsense mediated decay [11]. However the mutated cDNA was readily amplified from lymphocyte RNA, suggesting that the mutated mRNA is still present at a significant level.

It may be significant that the second affected sibling died of respiratory failure. LCA5 is known to be a ciliopathy, a disease resulting from a defect in formation or function of cilia. Cilia proteins are essential in the retina because the outer segments of rod and cone photoreceptors are highly adapted cilia. However, most human cells are ciliated and therefore the majority of these proteins would be expected to serve similar functions elsewhere in the body. To date all reported cases of LCA associated with mutations in or linkage to the *LCA5* gene lack other syndromic features. This is surprising as other ciliopathies affect the kidney and other organs, and the *LCA5* gene is known to be expressed in nasopharynx, trachea, and lungs and was originally identified in the proteome of bronchial epithelium ciliary axonemes [5]. If other LCA5 cases were found to have respiratory defects this might imply a defect of motor as well as sensory cilia in these patients.

In summary, this is the second report of *LCA5* mutations in LCA patients, further emphasizing the significance of mutations in this gene as a cause of LCA. The mutation identified is novel and causes disease by disrupting an existing splice donor site so that a cryptic donor site in the adjacent intron is favored, leading to a frameshift in the resultant mRNA.
